# Nox2 signaling and muscle fiber remodeling are attenuated by losartan administration during skeletal muscle unloading

**DOI:** 10.14814/phy2.14606

**Published:** 2021-01-05

**Authors:** Jeffrey M. Hord, Marcela M. Garcia, Katherine R. Farris, Vinicius Guzzoni, Yang Lee, Matthew S. Lawler, John M. Lawler

**Affiliations:** ^1^ Redox Biology & Cell Signaling Laboratory Department of Health and Kinesiology Graduate Faculty of Nutrition Texas A&M University College Station TX USA; ^2^ Department of Cellular and Molecular Biology Federal University of Paraíba João Pessoa, Paraíba Brazil; ^3^ Department of Systems Biology and Translational Medicine Texas A&M Health Science Center College of Medicine College Station/Temple TX USA; ^4^ Coulter Department of Biomedical Engineering Georgia Institute of Technology Atlanta GA USA; ^5^ Present address: Howard Hughes Medical Institute University of Iowa 4283 Carver Biomedical Research Building, 285 Newton Road Iowa City IA 52241‐1101 USA

**Keywords:** Angiotensin II type 1 receptor, hindlimb unloading, NADPH oxidase‐2, neuronal nitric oxide synthase, skeletal muscle atrophy

## Abstract

Reduced mechanical loading results in atrophy of skeletal muscle fibers. Increased reactive oxygen species (ROS) are causal in sarcolemmal dislocation of nNOS and FoxO3a activation. The Nox2 isoform of NADPH oxidase and mitochondria release ROS during disuse in skeletal muscle. Activation of the angiotensin II type 1 receptor (AT1R) can elicit Nox2 complex formation. The AT1R blocker losartan was used to test the hypothesis that AT1R activation drives Nox2 assembly, nNOS dislocation, FoxO3a activation, and thus alterations in morphology in the unloaded rat soleus. Male Fischer 344 rats were divided into four groups: ambulatory control (CON), ambulatory + losartan (40 mg kg^−1^ day^−1^) (CONL), 7 days of tail‐traction hindlimb unloading (HU), and HU + losartan (HUL). Losartan attenuated unloading‐induced loss of muscle fiber cross‐sectional area (CSA) and fiber‐type shift. Losartan mitigated unloading‐induced elevation of ROS levels and upregulation of Nox2. Furthermore, AT1R blockade abrogated nNOS dislocation away from the sarcolemma and elevation of nuclear FoxO3a. We conclude that AT1R blockade attenuates disuse remodeling by inhibiting Nox2, thereby lessening nNOS dislocation and activation of FoxO3a.

## Graphical abstract

Angiotensin receptor blockade (ARB) reduced unloading‐induced atrophy of skeletal muscle. Losartan mitigated assembly of the Nox2 complex, and thus oxidative stress ARB attenuated loss of sarcolemmal nNOSµ and nuclear levels of FoxO3a.

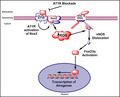

## 1 INTRODUCTION

Skeletal muscle is a highly dynamic tissue that responds to changes in mechanical loading by altering muscle fiber cross‐sectional area (CSA). Maintenance of muscle mass, fiber CSA, and contractile function are critical predictors of morbidity and mortality, as well as the quality of life. Muscle wasting is a characteristic of many acute and chronic diseases including the following: heart failure, cancer cachexia, chronic obstructive pulmonary disease, diabetes mellitus, neuromuscular diseases, and sarcopenia. In addition, prolonged periods of mechanical unloading or disuse such as forced bedrest, hospitalization, limb casting, and spaceflight elicit a loss of skeletal muscle fiber CSA, and thus a reduction in contractile force and mechanical power (Rejc et al., 2018).

Atrophy of muscle fibers during disuse is accomplished by altering protein metabolism and turnover, which is characterized by a reduction in protein synthesis coupled with an elevation of protein degradation (Schiaffino et al., 2013). Specifically, disuse or unloading downregulates Akt/mTORC1 anabolic signaling and increases activity of various proteolytic (e.g., calpains, caspases, ubiquitin‐proteasome pathway, autophagy‐lysosomal) pathways (Egerman & Glass, 2014; Schiaffino et al., 2013). A critical signaling event during disuse atrophy is the activation of forkhead members of the class O (FoxO) transcription factors (i.e., FoxO1/3a), and activation of downstream ubiquitin E3 ligases (e.g., muscle‐specific RING finger protein 1 (MuRF1), muscle atrophy F‐box (MAFbx)/atrogin‐1) (Bodine et al., 2001; Schiaffino et al., 2013).

The underlying cellular mechanisms that regulate unloading‐induced skeletal muscle fiber atrophy were canonically presumed to be multifactorial, but largely the reverse of homeostatic or hypertrophic pathways. However, new and emerging paradigms suggest instead that mechanosensing and nutrient sensing signaling serve as robust regulators of muscle fiber size, and therefore may be central to unloading‐induced atrophy. For example, ATF4 and p53 function as transcription factors to suppress spermine oxidase during short‐term disuse, promoting the expression of growth‐arresting proteins (Fox et al., 2014). A recent, paradigm‐changing event in mechanotransduction was the discovery that the mu splice variant of nitric oxide synthase (nNOSµ) migrates away from its perch on the dystrophin‐glycoprotein complex (DGC) to the cytoplasm in a variety of disuse models (Lawler et al., 2014; Llano‐Diez et al., 2012; Sandona et al., 2012; Suzuki et al., 2007). Indeed, translocation of nNOSµ away from the cell membrane into the cytoplasm is causal (Suzuki et al., 2007) in the dephosphorylation/activation of FoxO3a and muscle disuse and subsequent fiber atrophy (Lawler et al., 2014; Llano‐Diez et al., 2012; Sandona et al., 2012; Suzuki et al., 2007).

Prolonged periods of mechanical unloading are associated with increased production of reactive oxygen species (ROS) in skeletal muscle fibers (Kondo et al., 1993; Lawler et al., 2014). Moreover, the disuse‐induced elevation in oxidative stress can trigger atrophic signaling (Dodd et al., 2010). Our previous findings established, for the first time, a causal link between the disuse‐induced increase in ROS production and the translocation of nNOSµ away from the sarcolemma (Lawler et al., 2014). EUK‐134, a salen‐manganese mimetic of superoxide dismutase and catalase, prevented dephosphorylation of FoxO3a (Lawler et al., (2014)) and Akt (Kuczmarski et al., 2018).

Sources of ROS linked to disuse atrophy include the electron transport chain in mitochondria (Min et al., (2011); Talbert et al., 2013), xanthine oxidase (XO) (Derbre et al., 2012; Matuszczak et al., 2004), and NADPH oxidase (Nox) (Lawler et al., 2014). Two Nox isoforms have been identified so far in skeletal muscle: Nox2 (Lawler et al., 2014; Semprun‐Prieto et al., 2011) and Nox4 (Sun et al., 2011). Nox2 is a multi‐subunit complex that consists of two membrane‐bound subunits (gp91phox and p22phox) and four cytosolic subunits (p40phox, p47phox, p67phox, and Rac‐1). The Nox2 complex becomes active when the cytosolic subunits bind with the sarcolemmal subunits and convert molecular oxygen to the superoxide radical. Assembly of the Nox2 complex appears to be dependent on Rac‐1 activation, as well as protein kinase C (PKC)‐linked translocation of p47phox (Balakumar and Jagadeesh, (2014)).

We recently reported that 54 hr of hindlimb unloading was associated with increased sarcolemmal content of two Nox2 subunits, gp91phox and p47phox (Lawler et al., 2014). In addition, inhibition of Nox attenuated disuse‐induced contractile dysfunction and diaphragm muscle fiber atrophy in the rodent mechanical ventilation model (Llano‐Diez et al., 2012). Upstream signaling events involved in Nox2 assembly include angiotensin II (AngII) ligand‐binding of the AngII type 1 receptor (AT1R) which leads to phosphorylation of protein kinase C (PKC) (McClung et al., 2009). Exposure of skeletal muscle cells to elevated AngII levels results in increased oxidative stress and muscle atrophy (Kadoguchi et al., 2015; McClung et al., 2009; Semprun‐Prieto et al., 2011). However, the mechanotransductive properties of AT1R in unloading‐induced muscle fiber atrophy are unknown. Therefore, angiotensin II receptor blockade (ARB) was used to determine the causal role of AT1R on Nox2 activation, oxidative stress, and soleus muscle morphology (e.g., muscle fiber atrophy, partial fiber‐type shift from slow‐ to fast‐twitch) during mechanical unloading. The rodent hindlimb unloading model was utilized to induce mechanical unloading of hindlimb muscles. Thus, the hypothesis of this study was that intervention with the angiotensin receptor antagonist, losartan, would mitigate assembly of Nox2, elevated ROS, and thus translocation of nNOSµ, activation of FoxO3a, and alterations in soleus muscle morphology during unloading.

## 2 METHODS

### 2.1 Animals

All animal procedures were approved by the Institutional Animal Care and Use Committee (IACUC) at Texas A&M University. Young adult (4–5 months) male Fischer 344 (F344) rats were subjected to skeletal muscle disuse via hindlimb unloading. Animals were housed and cared for in accordance with the National Institutes of Health policy (NIH: DHEW publication no. 85‐23, revised 1985). Rat chow (4% fat) and water were provided ad libitum, and the animals were maintained in a temperature‐controlled room (23°C ± 2°C) with a 12:12 hr light–dark cycle. Rats are valuable models for the examination of skeletal muscle wasting that occurs during disuse and unloading in humans, with disuse‐induced responses such as changes in muscle fiber cross‐sectional area, weakness, and fiber‐type shifting from slow‐ to fast‐twitch, in a manner similar to human response (Edgerton et al., 1995; Fitts et al., 2010).

### 2.2 Hindlimb unloading

Systemic physiological effects of microgravity and bed rest are accurately replicated in the ground‐based hindlimb unloading model. An adaptation of the rodent tail‐traction hindlimb unloading model (Globus & Morey‐Holton, 2016) was utilized to induce mechanical unloading. Rats were anesthetized with an intraperitoneal (i.p.) injection of a ketamine (75 mg/kg)/xylazine (10 mg/kg) cocktail solution to induce unconsciousness, dampen sensation, and produce muscle relaxation, allowing research personnel to harness the tail. Tail harnesses were then connected to a cross‐wire that spanned across the top of the cage. The hindlimbs of the rats were lifted so that their hindfeet were approximately 1 cm off of the cage floor. Hindlimbs of the rats remained unloaded for a total of 7 days. At the end of the 7‐day period, rats were sacrificed. Rats were euthanized with 120 mg/kg i.p. sodium pentobarbital (Euthanasia III Solution).

### 2.3 Experimental design

A hindlimb unloading duration was chosen to target the early stages of skeletal muscle remodeling during disuse. Markers of atrophy and oxidative stress have been observed to peak or be near a peak state at the 7‐day time point during disuse (Dupont et al., 2011). The United States Food and Drug Administration (FDA)‐approved angiotensin II type 1 receptor blocker, losartan, as an intervention to target the potential upstream trigger of oxidative stress during disuse. Adult F344 rats were divided into four groups (*n* = 7/group): loaded controls (CON), 7 days of hindlimb unloading (HU), 7 days of HU + 40 mg kg^−1^ day^−1^ i.p. of losartan (HUL), and loaded controls + losartan (CONL). Losartan dosage used in this study was per the Burks et al. (2011) study. CON and HU rats received daily i.p. injections of similar volumes of physiological saline. Administration of losartan or saline began 24 hr prior to hindlimb unloading and continued throughout the 7‐day unloading period (8 days total). Sample size calculations were based on soleus muscle fiber cross‐sectional area, which has the lowest mean percentage difference among our results in preliminary studies.

### 2.4 Skeletal muscle tissue preparation

The soleus muscle was chosen as a model of skeletal muscle response to mechanical unloading disuse. As a postural muscle with a high percentage of Type I (slow twitch) fibers, the soleus is susceptible to rapid atrophy and remodeling when exposed to mechanical unloading (Ohira et al., 1972). Rats were euthanized with 120 mg/kg of pentobarbital sodium salt (Euthanasia III Solution) by way of an i.p. injection. The soleus muscles were extracted, trimmed of excess tissue, rinsed with phosphate‐buffered saline (PBS) solution and laid longitudinally on a polyurethane mount of tissue freezing medium before being frozen in liquid nitrogen‐cooled isopentane (2‐methylbutane). Samples were subsequently stored at −80°C until needed for the following experimental techniques or assays.

### 2.5 Subcellular fractionation

Soleus muscles were minced, weighed, and washed with cold PBS prior to homogenization. Subcellular fractionation was performed using adaptations of the methods described by Brenman et al. (1995) and Dimauro et al. (2012). Soleus samples were homogenized in lysis *Buffer A* (10:1 w/v) containing the following: 25 mM Tris‐HCL, pH 7.4, 100 mM NaCl, 1 mM ethylenediaminetetraacetic acid (EDTA), 1 mM ethylene glycol‐bis(b‐aminoethyl ether)‐N,N,N',N'‐tetraacetic acid (EGTA), and protease inhibitor cocktail. Muscles were homogenized using a motorized ground glass‐on‐ground glass mortar and pestle at 4°C. Nuclei and debris were pelleted by centrifugation at 1,000*g* for 10 min. The resulting supernatant was then centrifuged at 20,000*g*. The resulting supernatant was the cytoplasmic fraction while the remaining pellet was resuspended in *Buffer A* and deemed the crude membrane fraction.

The nuclear fraction was isolated as described by Dimauro et al. (2012) with minor modifications. Nuclei and debris pelleted following centrifugation at 1,000*g* (as noted in the previous paragraph) was resuspended in *Buffer B* (6:1 w/v) which contained 250 mM sucrose, 50 mM Tris‐HCL pH 7.4, 5 mM MgCl_2_, and protease inhibitor cocktail, vortexed and then centrifuged at 500*g* for 15 min. The resulting supernatant was discarded and the pellet was resuspended in *Buffer B*, vortexed and then centrifuged at 1,000x*g* for 15 min. Once again, the supernatant was discarded and the pellet was resuspended in *Buffer C* (20 mM HEPES pH 7.9, 1.5 mM MgCl_2_, 0.5 M NaCl, 0.2 mM EDTA, 20% glycerol, 1% Triton‐X‐100, protease inhibitor cocktail). This fraction was then incubated on ice for 30 min and intermittently vortexed. Next, the fraction was centrifuged at 9,000*g* for 30 min, and the resulting supernatant was deemed the nuclear fraction.

Protein concentrations were determined using the Bradford protein assay, following the manufacturer's instructions (Bio‐Rad, cat # 500–0006). Tissue extracts were subsequently aliquoted and stored at −80°C until western blot analysis. Validation of each subcellular fraction was assessed by western blotting for GAPDH (cytoplasmic) (1:10,000, EMD Millipore, cat # MAB374), Na^+^/K^+^ ATPase α‐1 (crude membrane) (1:1,000, EMD Millipore, cat # 05‐369), and Histone H3 (nuclear) (1:1,000, Cell Signaling Technology, 9715).

### 2.6 Western Immunoblotting

Soleus muscle extracts (20 µg or 30 µg) along with sample buffer were loaded into wells of 8% or 10% sodium dodecyl sulfate‐polyacrylamide gel electrophoresis gels. Electrophoresis was conducted at 120 V for ~75 min. Gels were then transferred at 100 V for ~60 min onto a nitrocellulose membrane (Bio‐Rad, cat # 162‐0112). Membranes were blocked in a nonfat milk buffer (5% nonfat milk in TBS) for 1 hr. Following blocking, membranes were incubated overnight (4°C) in blocking buffer with the appropriate primary antibody: anti‐nNOS (1:750, Life Technologies, cat # 61–700), anti‐gp91phox (1:1,000, BD Biosciences, cat # 611415), anti‐p67phox (1:2,500, BD Biosciences, cat # 610913), anti‐p47phox (1:750, BD Biosciences, cat # 610355), anti‐Rac‐1 (1:500, EMD Millipore, cat # 07‐1464), anti‐FoxO3a (1:750, Cell Signaling Technology, cat # 12829), and anti‐p53 (1:750, Cell Signaling Technology, cat # 2527). Membranes were subsequently washed in Tris buffered saline (TBS) with 0.1% Tween‐20 (TBS‐T) (3 × 5 min) and then incubated at room temperature for 1 hr in blocking buffer containing the appropriate horseradish peroxidase‐conjugated secondary antibodies (Santa Cruz Biotechnology, cat # sc‐2004 or sc‐2005). After TBS‐T washes, proteins were visualized by Super Signal West Dura Extended Duration Substrate (Thermo Scientific, cat # 34076) enhanced chemiluminescence detection, and developed with the Fuji LAS‐3000 Luminescent Image Analyzer (FujiFilm Medical Systems). Quantification was performed using NIH ImageJ software. Ponceau S staining (cytoplasmic: band ~38 kDa mark (GAPDH); crude membrane: band ~42 kDa mark (actin); nuclear: band ~42 kDa mark (actin)) was used as a loading control.

### 2.7 Histological analysis

Soleus muscles were embedded in tissue freezing medium and subsequently frozen. Muscles were cut in 10‐µm sections from the midbelly of the muscle using a cryostat (Thermo Scientific, Shandon Cryotome FSE) and allowed to air‐dry for 30 min. Hematoxylin and eosin (H&E) stains were used to assess tissue morphology and performed as previously described (Lawler et al., 2014).

Muscle sections were also stained with the wheat germ agglutinin (WGA) Alexa Fluor 555 conjugate (WGA‐Alexa 555, 1:40, Life Technologies, cat # W32464) in order to visualize the sarcolemma and connective tissue. Sections were costained with a 4',6‐diamidino‐2‐phenylindole (DAPI) solution (1:500, Life Technologies, cat # D1306) for 5 min according to the manufacturer's guidelines. Slides were air‐dried prior to coverslip mounting with Prolong Gold anti‐fade medium (Life Technologies, Grand Island, NY).

NADPH‐diaphorase histochemical analysis was used to examine the presence and localization of active nNOS. This assay was performed as previously described (Vitadello et al., 2014) with minor modifications. Ten‐micrometer thick soleus muscle sections were fixed for 20 min with 2% paraformaldehyde followed by a PBS wash. Sections were then incubated in a buffer containing 50 mM Tris‐HCl pH 8.00, 0.2% Triton‐X‐100, 0.5 mM nitrotetrazolium blue chloride, and 1 mM β‐NADPH for 2 hr at 37°C. The enzymatic reaction was stopped by briefly rinsing the slides with distilled water and then allowed to air‐dry. Coverslips were mounted onto the samples with permanent mounting medium (Vectamount, Vector Laboratories, cat # H‐5000).

Stained sections were visualized and photographed with a Zeiss Axioplot upright microscope and Zeiss Axiocam HRc color camera (Carl Zeiss Microimaging, Thornwood, NY). NADPH‐diaphorase stained samples (*n* = 5–6/group) were quantified by using NIH ImageJ to obtain the cross‐sectional circumference (CSC) and determining the percentage of the sarcolemma that stained positively for NADPH‐diaphorase.

### 2.8 Immunofluorescence

To examine protein localization, soleus muscle cross‐sections obtained from the midbelly were serially sectioned at 10 µm thickness in a cryostat at −15°C and placed onto microscope slides. Samples were fixed in either acetone (myosin heavy chain immunofluorescence) at −20°C for 10 min or in 2% paraformaldehyde followed by a 20‐min incubation in citrate buffer at 92°C (nNOS and FoxO3a immunofluorescence). Following fixation, sections were washed in PBS with 0.1% Tween‐20 (PBS‐T). Sections were blocked in a 10% normal goat serum (Thermo‐Fisher Scientific, cat # 50062Z) for 15 min. After blocking, sections incubated in blocking buffer containing specific primary antibodies: dystrophin (1:100, Santa Cruz Biotechnology cat # sc‐15376), slow skeletal myosin heavy chain (1:250, Abcam, cat # ab11083), fast skeletal myosin heavy chain (1:250, Abcam, cat # ab51263), nNOS (1:100, Cayman Chemical, cat # 160870), FOXO3a (1:50, Sigma‐Aldrich, cat # SAB3500508), and Beta‐Sarcoglycan (1:200, Abcam, cat # ab55683) for 1 hr in an enclosed chamber at room temperature. After three 5‐min washes in PBS, sections were incubated in the appropriate secondary antibody with a fluorophore attached (e.g., goat anti‐rabbit Alexa Fluor 488, goat anti‐mouse Alexa Fluor 594) (1:200) for 30 min at room temperature. Sections were subsequently washed twice in PBS‐T and once in PBS. Some instances required DAPI staining, which involved 5 min incubation followed by PBS washes. Slides were allowed to air‐dry prior to mounting with Prolong Gold anti‐fade medium (Life Technologies, Grand Island, NY). Images were captured on a Zeiss Axioplot upright microscope and Zeiss Axiocam HRc color camera.

Muscle fiber CSA was determined on β‐sarcoglycan‐ and dystrophin‐stained muscle cross‐sections by employing the semiautomatic muscle analysis using segmentation of histology (SMASH) MATLAB‐based program (Smith & Barton, 2014). A range of 200–250 fibers per muscle were quantified to obtain an average CSA from each sample (*n* = 7/group). Quantification of the percentage of FoxO3a‐positive myonuclei was accomplished by triple staining soleus sections with anti‐FoxO3a, anti‐β‐Sarcoglycan, and DAPI to determine the colocalization of FoxO3a with myonuclei. A minimum of 300 myonuclei were counted per muscle sample (*n* = 4/group) to obtain the percentage of FoxO3a‐positive myonuclei.

### 2.9 Oxidative stress marker & serum measurements

ROS generation was determined via a dihydroethidium (DHE) fluorescence protocol on soleus cross‐sections. DHE is oxidized by superoxide to produce a fluorescent ethidium. Soleus muscle sections (10 µm) were air‐dried and rehydrated with PBS. Sections then incubated with 5 µmol DHE (Life Technologies, cat # D1168) in a dark, humidified chamber for 30 min at 37°C. Muscle sections were washed with PBS, air‐dried and mounted with ProLong Gold Anti‐fade mounting medium. Immediately after mounting the coverslips, images taken at 10× magnification were obtained using the Zeiss Axioplot microscope and HRc color camera. Ethidium‐positive nuclei were quantified using particle analysis functions on NIH ImageJ. Four images taken at 10x magnification were quantified per sample (*n* = 6).

NADPH‐dependent superoxide production was assessed with the lucigenin chemiluminescent assay (Heymes et al., 2003; Whitehead et al., 2010). Soleus muscle tissues were pooled together (*n* = 3 per pool) and homogenized in ice‐cold buffer containing 25 mM Tris‐HCL, pH 7.4, 100 mM NaCl, 1 mM EDTA, 1 mM EGTA, and a protease inhibitor cocktail. Protein concentrations of the total homogenate were determined using Bradford assay. Experiments were performed on a microplate luminometer (BioTek Synergy 4 Microplate Reader; 37°C) using 100 µg of protein per well, NADPH (300 µM) and lucigenin (10 µM). In order to confirm that the signal was due to superoxide production, a superoxide dismutase mimetic (EUK‐134, 50µM, Cayman Chemical, cat # 10006329) was used to scavenge superoxide. In addition, pharmacological inhibitors were used to confirm that the activity was primarily due to NADPH oxidase: the nonspecific NADPH oxidase inhibitor diphenyleneiodonium (DPI, 10 µM, Sigma‐Aldrich, cat # D2926) and the selective (Csanyi et al., 2011) Nox2 inhibitor gp91ds‐tat (5 µM, BioSynthesis, Inc., Lewisville, TX).

Serum angiotensin II was measured using a Thermo‐Fisher ELISA kit. The protocol was performed as suggested by the manufacturer. Serum samples were pooled together (*n* = 3 per pool) and run in duplicate. Values were expressed as a percent of positive controls.

### 2.10 Statistical design

All data reported here are as mean ± *SEM*. Statistical analysis was performed using GraphPad 6 software (GraphPad Software, Inc. San Diego, CA). One‐way ANOVA was used to identify differences among CON, HU, HUL, and CONL. Tukey's post hoc analysis was utilized when necessary. Significance level for all tests was set at *p* < .05.

## 3 RESULTS

### 3.1 Hindlimb unloading‐induced soleus muscle remodeling is mitigated by AT1R blockade

We initially tested the hypothesis that AT1R blockade would ameliorate the hindlimb unloading‐induced mass and morphological alterations in rat soleus muscles. An isolated report found that AT1R blockade offers protection against limb‐stapling‐induced atrophy in aging, sarcopenic mice (Burks et al., 2011). Moreover, our research group recently discovered that Nox2 inhibition reduces muscle fiber atrophy during unloading (Ryan et al., 2017). However, it was unknown whether AT1R blockade mitigates unloading‐induced fiber atrophy in young, adult rats. In this study, seven days of hindlimb unloading resulted in a significant drop in body weight compared to the control group (287.1 vs. 342.1 g; *p* = .003). Administration of losartan during HU did not offer significant protection against the small HU‐induced reduction in body weight (299 vs. 287.1 g; *p* = .826). In addition, soleus muscles from HU rats were significantly smaller than those from controls (103 vs. 123.1 mg; *p* = .001). Soleus muscles from HUL rats were not significantly protected from the HU‐induced drop in muscle weight (106.8 vs. 103 mg; *p* = .8687). Ambulatory control rats that received losartan (CONL) did not differ from CON rats in body weight (*p* = .999) or soleus weight (*p* = .9043).

**FIGURE 1:**
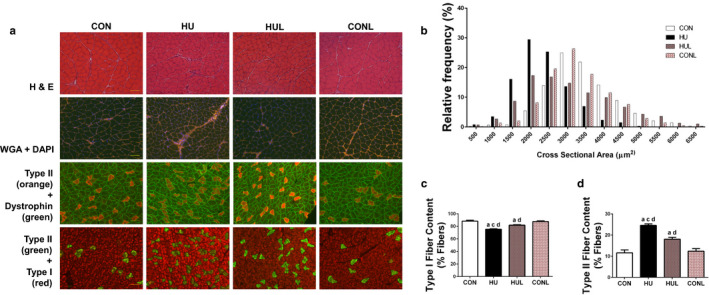
AT1R blockade mitigates soleus muscle fiber remodeling due to one week of hindlimb unloading. A (top row), representative images of hematoxylin & eosin (H&E) stained soleus muscles; (second row), representative images of muscles stained for wheat germ agglutinin (WGA) and DAPI. (third row), representative immunofluorescence images of soleus muscles stained for type II muscle fibers (orange) and dystrophin (green); (bottom row), images of soleus muscles stained for type II muscle fibers (red) and type I muscle fibers (green). Scale bar = 100 µm. B, Distribution of muscle fibers according to fiber cross‐sectional area (CSA) for controls (CON), hindlimb unloaded (HU), hindlimb unloaded + Losartan (HUL), and control + Losartan (CONL) *(n* = 7/ group). C, percentage of soleus muscle fibers that are type I fibers. D, percentage of muscle fibers that are type II fibers. Values are means ± *SEM*. Letters indicate groups are significantly different from each other (*p* < .05): ^a^indicates different from CON; ^c^indicates different from HUL; ^d^indicates different from CONL

H&E, WGA, and fiber‐type stains are illustrated in Figure 1a. Quantification of the average CSA of the soleus muscles revealed that unloading resulted in a significant reduction of 34% in myofiber CSA compared to ambulatory controls (3,469 µm^2^ vs. 2,291 µm^2^; *p* < .0001). However, losartan administration offered significant protection against unloading‐associated reduction in fiber CSA (2,996 µm^2^ vs. 2,291 µm^2^; *p* = .001), but still remained significantly smaller than CON (*p* = .0328). The majority of fibers in HU muscles were in the 1,000–3,000 µm^2^ range (84.5%), whereas 83.8% of the CON fibers were found in the 2,000–4,500 µm^2^ range. Fibers from the HUL muscles were predominately in the 1,000–4,500 µm^2^ range (85.9%), exhibiting a wider range of fiber CSA than the HU muscles (Figure 1b). In addition, fiber CSA from CONL rats did not differ from CON rats in fiber CSA, illustrating that the protective effects of losartan were limited to unloaded rats.

Additionally, mechanical unloading‐induced shift of soleus muscle fiber type from slow‐ to fast‐twitch (i.e., Type I to Type II) was partially abrogated by AT1R blockade. Representative immunofluorescent fiber‐type specific stains are shown in Figure 1a. As expected, unloading resulted in a significant reduction in the percentage of Type I fibers compared to CON (75.41% vs. 88.34%; *p* < .0001) (Figure 1c), concomitant with a significant increase in the percentage of Type II fibers (24.59% vs. 11.66%; *p* < .0001) (Figure 1d). These effects were mitigated with losartan administration; however, HUL soleus muscle fiber‐type composition still remained significantly different from CON, with a 6.46% difference (*p* = .0015) (Figure 1c and 1d). Soleus muscle fiber‐type composition did not differ between CON and CONL rats.

### 3.2 Increased ROS production during hindlimb unloading is partially prevented by losartan treatment, and linked to suppression of active Nox2

**FIGURE 2:**
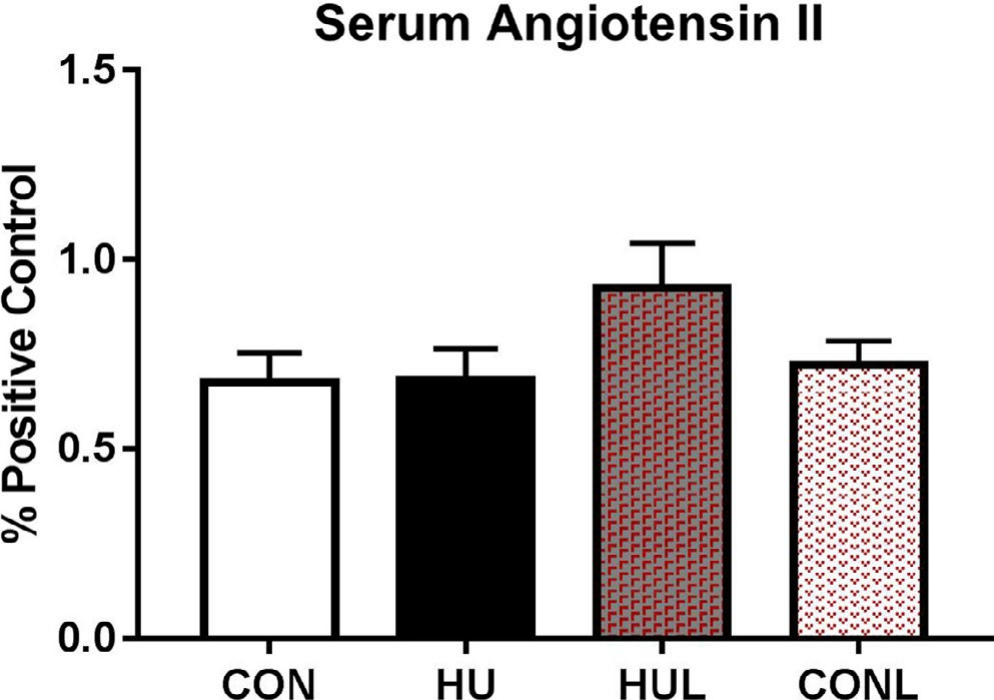
Serum angiotensin II levels as expressed as % of positive controls. Experimental groups included controls (CON), hindlimb unloaded (HU), hindlimb unloaded + Losartan (HUL), and control + Losartan (CONL) *(n* = 7/group), No significant effects for hindlimb unloading or losartan intervention were found

In the next set of experiments, we tested the hypothesis that hindlimb unloading‐induced production of reactive oxygen species would be reduced, primarily via Nox2 inhibition, by daily losartan administration. Firstly, serum angiotensin II levels were tested in order to determine if angiotensin II was upregulated during unloading and whether losartan affected the concentration of angiotensin II (Figure 2). As expressed per % of positive control, surprisingly, seven days of hindlimb unloading had no elevating effect on serum [AngII]. While the losartan‐treated HU group demonstrated a 25% trend upwards, it was not significantly different from the HU mean (*p* = .1917).

**FIGURE 3:**
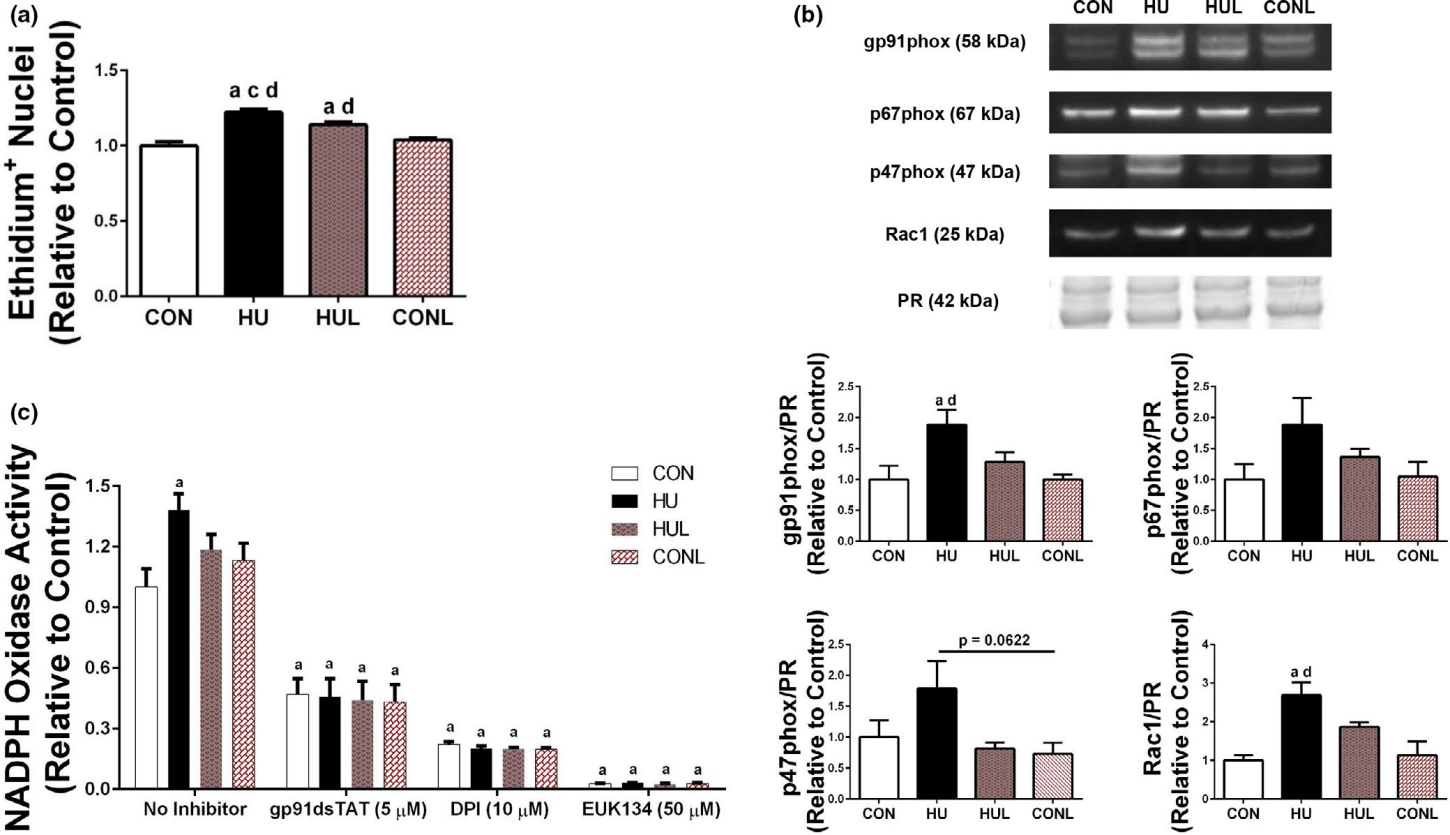
Hindlimb unloading leads to a rise in superoxide production. A, quantification of ethidium‐positive nuclei from soleus muscle sections that were incubated with dihydroethidium (DHE) to indicate superoxide production. Scale bar = 100 µm. B, representative western blot detection of select Nox2 subunits in the soleus muscle membrane fraction. Quantification of Nox2 western blot detection. Ponceau S Red (PR) stain (~42 kDa mark) used as a loading control for western blotting quantification. C, NADPH‐dependent superoxide production was assessed by lucigenin chemiluminescence. Soleus muscles were pooled for each group (2 pools per group; *n* = 3 muscles per pooled sample). Comparison of superoxide production in the absence and presence of ROS inhibitors: EUK‐134 (50 µM), an SOD/Catalase mimetic; diphenyleneiodonium (DPI; 10 µM), a nonspecific NADPH oxidase inhibitor; and gp91ds‐tat (5 µM), a NADPH oxidase isoform 2 specific, peptidyl inhibitor. Superoxide production was compared to the level of control without an inhibitor. Values are presented as fold control of mean ± *SEM*. Values are presented as fold control of mean ± *SEM*. Letters indicate groups are significantly different from each other (*p* < .05): ^a^indicates different from CON; ^c^indicates different from HUL; ^d^indicates different from CONL

Soleus muscle cross‐sections were incubated with dihydroethidium (DHE) to assess superoxide production. A significant rise in the number of nuclei positively stained with ethidium in HU cross‐sections compared to the CON muscles (*p* < .0001) (Figure 3a) was observed. In addition, significant protection offered by losartan treatment during unloading (*p* = .0416). However, HUL still contained a significantly greater amount of ethidium‐positive nuclei compared to CON and CONL (*p* = .0001 and *p* = .0091, respectively).

Given that the hindlimb unloading‐induced elevation in superoxide production was mitigated by losartan treatment, and previous findings showed that AT1R blockade ameliorates NADPH oxidase accumulation and production of superoxide, the next step was to measure the protein content of Nox2 subunits in the membrane fraction of the soleus muscle fibers. Western blot experiments showed a significantly greater content of the membrane‐bound gp91phox Nox2 subunit in HU samples compared to CON (*p* = .0105) (Figure 3b). This effect was prevented by losartan treatment during HU, with only a 28% rise in gp91phox content compared to CON, while remaining 60% lower than HU (*p* = .1192). Examination of sarcolemma‐localized content of the transportable, cytosolic subunits showed consistently increased trends among p67phox and p47phox subunits in HU compared to CON (79% and 88.7% increases, respectively) (Figure 3b). In addition, a significant increase in sarcolemma content of Rac‐1 was observed in HU samples versus CON (*p* = .001) (Figure 3b). Losartan partially prevented the rise in protein abundance of sarcolemma‐localized Nox2 subunits, with each of the measured subunits in the HUL group remaining nonsignificantly different from CON and CONL groups.

Given that unloading increased protein content of sarcolemma‐localized Nox2 subunits, and the implication of elevated Nox2 activity, we tested whether Nox2 complex assembly during hindlimb unloading led to elevated Nox2 activity. NADPH‐dependent superoxide production was measured by lucigenin chemiluminescence in soleus muscle homogenates. Using NADPH as a substrate, superoxide was significantly increased by 38.4% in HU muscles compared to CON (*p* = .0282) (Figure 3c). In order to establish that NADPH oxidase was a primary source of NADPH‐dependent superoxide production in the HU soleus muscles, inhibitors of NADPH oxidase and a potent superoxide dismutase mimetic (EUK‐134) were used. Treatment with 50 µM of EUK‐134 resulted in an almost complete abolishment of superoxide production. The nonspecific NADPH oxidase inhibitor diphenyleneiodonium (DPI) prevented approximately an 80% reduction of superoxide production across the groups, strongly suggesting that the superoxide produced was primarily due to NADPH oxidases. Use of the Nox2‐specific peptidyl inhibitor gp91ds‐tat resulted in more than 50% reduction in superoxide production, suggesting Nox2 as a primary Nox complex contributing to NADPH‐dependent superoxide production.

### 3.3 Dislocation of sarcolemmal nNOSµ with prolonged mechanical unloading is mitigated by AT1R blockade

**FIGURE 4:**
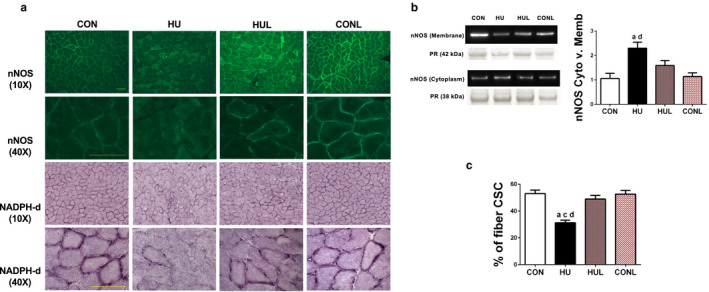
Unloading‐induced reduction of sarcolemmal neuronal nitric oxide synthase (nNOS) content in soleus muscle fibers is mitigated by losartan treatment. A (top row, second row), representative immunofluorescent images of nNOS stained soleus muscle sections at 10× and 40×. (third row), representative images of soleus muscle sections subjected to NADPH‐diaphorase (NADPH‐dia) histochemical assay to detect sarcolemmal nNOS activity taken at 10× magnification; (bottom row), NADPH‐dia images taken at 40× magnification. Scale bar = 100 µm. Scale bar = 100 µm. B, western blot detection of nNOS protein content in the membrane fraction and the cytosolic fraction. Ratio of cytosolic nNOS content to membrane content of nNOS based subcellular fractionation is presented in the accompanying graph. C, quantification of NADPH‐dia reactive staining found at the sarcolemma. Presented as percentage of cross‐sectional circumference (CSC) that is stained positive. Ponceau S Red (PR) stain (~42 kDa mark for membrane fraction; ~38 kDa mark for cytosolic fraction) used as a loading control for western blotting quantification. Values are presented as fold control of mean ± *SEM*. Values sharing the same letter are not significantly different (*p* < .05): ^a^indicates different from CON; ^c^indicates different from HUL; ^d^indicates different from CONL

Next, the hypothesis that hindlimb unloading‐induced dislocation of nNOSµ from the sarcolemma would be protected by AT1R blockade was tested. Localization of nNOSµ protein was assessed by immunofluorescence staining which revealed a substantial reduction in sarcolemma‐localized nNOSµ in HU soleus muscle fibers, an effect that appeared to be partially prevented by treatment with losartan (Figure 4a). To confirm the immunofluorescence observations, subcellular fractionation and western immunoblotting was performed to detect membrane‐localized and cytoplasmic localized nNOS protein abundance (Figure 4b). HU muscles were found to have 52.9% less sarcolemma‐localized nNOSµ than CON (*p* < .0001), whereas HUL muscles contained only 26.5% less (*p* < .0001), although still significantly lower than CON and CONL (*p* = .0121 and 0.0032, respectively). While cytoplasmic nNOS was not found to be different across groups, there was a 30% increase in cytoplasmic nNOS in both the HU and HUL groups compared to CON, and a 20% increase in CONL compared to CON. The ratio of cytoplasmic: membrane nNOS was significantly elevated in the HU group compared to CON (*p* = .0029), whereas the ratio in HUL soleus was not found to be significantly different from CON or CONL (Figure 4b).

Examination of whether or not the displacement and reduction of sarcolemma nNOSµ signified an alteration in nNOS‐produced •NO was performed next. Soleus muscle cross‐sections were subjected to NADPH‐diaphorase staining to determine the localization of active •NO in muscle fibers, as seen in Figure 4a. Visually, apparent differences in sarcolemma nNOSµ activity were easily noticeable between CON and HU. Determination of the percentage of NADPH‐diaphorase‐positive staining along the sarcolemma revealed a similar decrement in nNOSµ activity in HU versus CON (*p* < .0001) (Figure 4c) as that found in the nNOSµ membrane localization experiments. Interestingly, losartan administration during HU maintained sarcolemma‐associated nNOSµ activity near control levels (49% versus 52.96%; *p* = .6897), which was dramatically higher than HU (*p* < .0001) (Figure 4c).

### 3.4 AT1R blockade significantly lessens the unloading‐induced nuclear accumulation of FoxO3a

**FIGURE 5:**
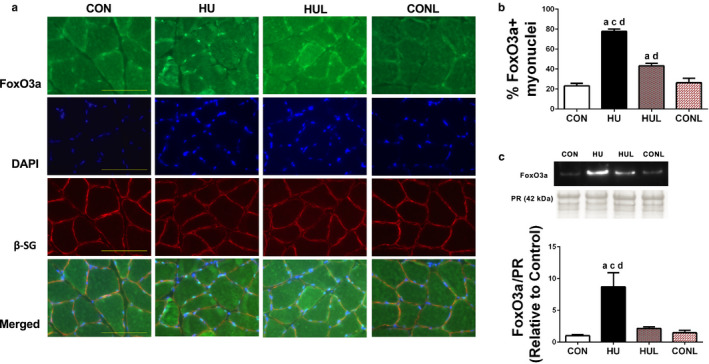
Nuclear localization of FoxO3a due to hindlimb unloading disuse is partially prevented by losartan administration. A, soleus muscle cross‐sections stained with (top row) FoxO3a, (second row) DAPI, (third row) beta‐sarcoglycan (b‐SG), and the bottom row is comprised of the merged images. Scale bar = 100 µm. B, graphical representation of the percentage myonuclei positively stained for FoxO3a. Values are means ± *SEM*. C, detection of FoxO3a via western blotting in the nuclear fraction. Ponceau S Red (PR) stain (~42 kDa mark) used as a loading control for western blotting quantification. Values are presented as fold control of mean ± *SEM*. Values sharing the same letter are not significantly different (*p* < .05): ^a^indicates different from CON; ^c^indicates different from HUL; ^d^indicates different from CONL

Important regulators of skeletal muscle atrophy with disuse include FoxO3a and p53 transcription factors. Our laboratory and other investigators have previously shown that dephosphorylation and activation of FoxO3a was dependent on nNOSµ dislocation from the sarcolemma (Lawler et al., 2014; Suzuki et al., 2007). Given that losartan mitigated nNOSµ translocation and alterations in sarcolemmal nNOSµ activity, the next aim was to determine if losartan would lead to a reduction in nuclear FoxO3a. Colocalization of positively stained FoxO3a myonuclei with DAPI demonstrated a dramatic rise (> 50%) in nuclear‐localized FoxO3a in HU soleus versus CON (*p* < .0001) (Figure 5a and b). This effect was ameliorated in HUL muscles with only a 20% increase versus CON, and 45% lower than HU (*p* < .0001). However, HUL muscles still contained a significantly greater amount of positively stained FoxO3a nuclei compared to CON and CONL (*p* = .6897 and 0.742, respectively). Further confirmation of hindlimb unloading‐induced nuclear translocation of FoxO3a was observed with western blotting of the nuclear fraction (Figure 5c). HU displayed a dramatic 770% increase in FoxO3a nuclear content compared to CON (*p* = .0003). HUL remained 655% lower than HU in FoxO3a nuclear content (*p* = .0019). In contrast, blot data did not find a significant elevation in FoxO3a nuclear content among HUL, CON and CONL groups.

**FIGURE 6:**
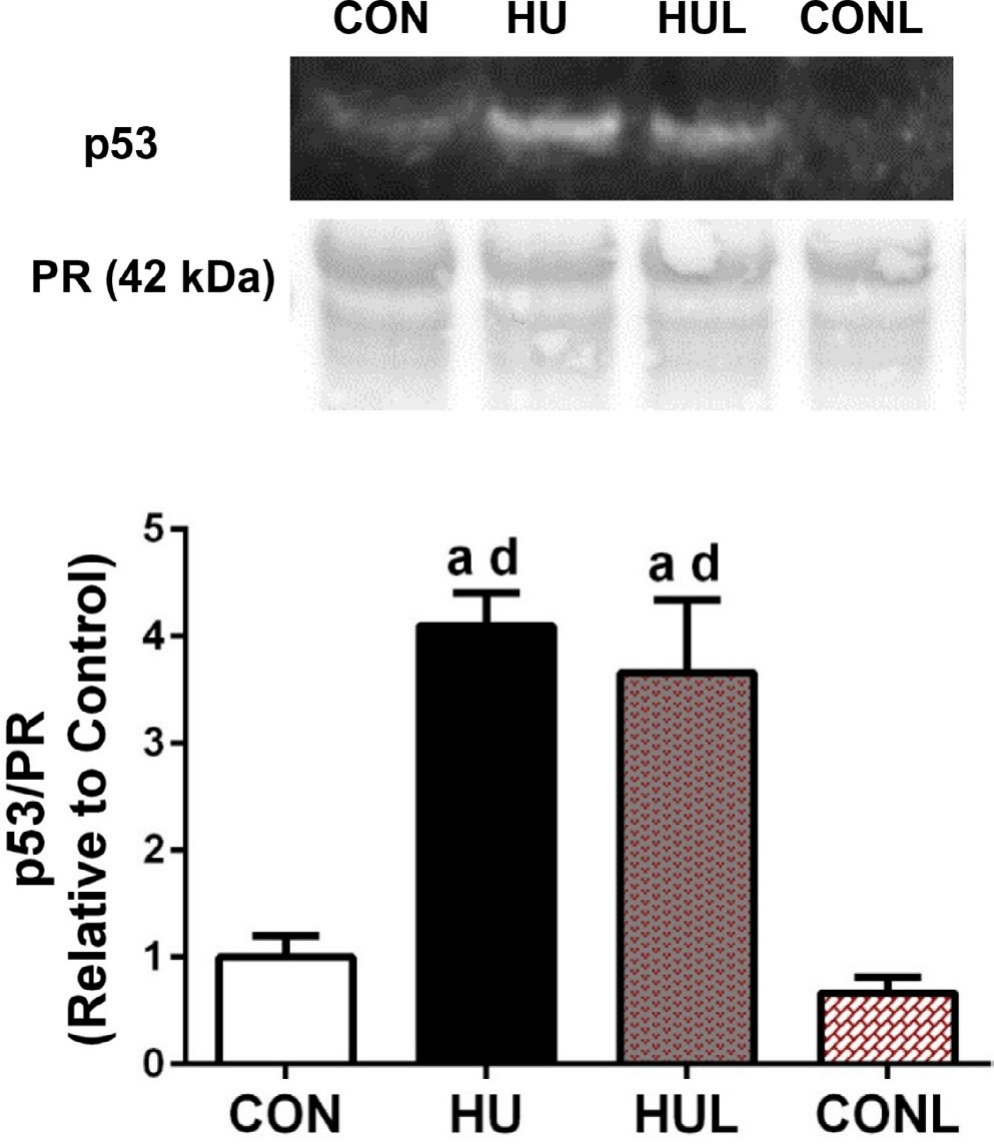
Nuclear content of the atrophy‐inducing transcription factor p53 is dramatically increased following 7 days of hindlimb unloading, even in losartan‐treated animals. Western blot detection of p53 in the nuclear fraction of soleus muscles. Ponceau S Red (PR) stain (~42 kDa mark) used as a loading control for western blotting quantification. Values are presented as fold control of mean ± *SEM*. Values sharing the same letter are not significantly different (*p* < .05): ^a^indicates different from CON; ^c^indicates different from HUL; ^d^indicates different from CONL

In addition to FoxO3a, p53 has been found at increased concentrations and activity levels as a transcription factor during the early stages of disuse (Fox et al., 2014). Therefore, the nuclear fraction of soleus muscles was probed to assess p53 protein content. Similar to the hindlimb unloading‐induced increase in FoxO3a nuclear content, p53 nuclear content was significantly increased 310% in HU muscle compared to CON (*p* < .0001) (Figure 6). In contrast to the FoxO3a nuclear content, HUL muscles were found to have a significant elevation of nuclear p53 compared to CON and CONL (*p* = .0003 and *p* < .0001, respectively). These data indicate that losartan may be selectively interfering with atrophic pathways with mechanical unloading, independent of p53 signaling.

## 4 DISCUSSION

In this study, administration of the angiotensin II type 1 receptor (AT1R) blocker losartan during hindlimb unloading attenuated the disuse‐induced phenotypic alterations: reductions in soleus muscle fiber CSA and the slow‐twitch to fast‐twitch fiber‐type shift. These effects were linked directly to abrogation of unloading‐induced accumulation and activation of Nox2, thus mitigating a rise in ROS levels. In addition, losartan treatment during mechanical unloading significantly mitigated unloading‐induced dislocation of nNOS from the sarcolemma. Furthermore, losartan treatment also reduced the nuclear accumulation of the atrophy‐associated transcription factor FoxO3a, which has been previously observed (Suzuki et al., 2007; Vitadello et al., 2014) to be activated by translocation of nNOS away from the sarcolemma. The following paragraphs provide a discussion of the principle findings.

Losartan treatment has previously been shown to prevent the reduction in myofiber CSA following 21 days of limb immobilization in aged mice (Burks et al., 2011). Our findings indicate that hindlimb unloading‐induced reduction in myofiber CSA and fiber‐type shift (Figure 1) were limited by daily administration of losartan following one week of unloading. Importantly, soleus muscle remodeling in response to mechanical unloading was not linked to changes in serum angiotensin II levels (Figure 2). Furthermore, the ameliorating effects of losartan were not related to a decrease in serum [AngII] levels. Interestingly, Ang II‐independent mechanical activation of AT1Rs is known to contribute to the regulation of myogenic tone in resistance arteries (Blodow et al., 2014; Hong et al., 2016, 2017; Pires et al., 2017; Schleifenbaum et al., 2014). Pharmacological AT1R antagonism via losartan or candesartan is also associated with a significant reduction in ligand‐independent AT1R mechano‐signaling and myogenic vasoconstriction. Therefore, we postulate that AT1R may trigger assembly of Nox2 and ROS production via a mechanosensing response in unloaded skeletal muscle, independent of a rise in angiotensin II levels.

While losartan administration offered partial protection against the unloading‐induced decrease in muscle fiber CSA and fiber‐type shift, it did not protect against loss of overall soleus mass. This finding may be explained in part by the impact of AT1R blockade on the prevention of extra‐myocyte build‐up in the soleus muscle. In support of this observation, AT1R antagonism via losartan ameliorates skeletal muscle fibrosis and extra‐myofiber accumulation due to disuse, injury, (Burks et al., 2011) and CTGF overexpression (Cabello‐Verrugio et al., 2012). Interestingly, Ang‐(1‐7), a downstream antagonist fragment of AngII produced via ACE2 (angiotensin converting enzyme II), inhibited casting‐induced reduction of fiber CSA in the tibialis anterior following 2 weeks of immobilization, and was dependent upon the G‐protein Mas receptor (Morales et al., 2016). In summary, daily losartan treatment during unloading attenuated critical aspects of the morphological remodeling associated with prolonged disuse, even though total soleus mass remained lower.

Data presented here are consistent with the hypothesis that elevated ROS contributes to disuse‐induced translocation of nNOSµ from the sarcolemma and muscle fiber remodeling. An unloading‐induced rise in superoxide anions, sarcolemmal assembly of Nox2 subunits, and Nox2 activation were all observed (Figure 3). Indeed, increased content of the membrane‐bound subunit gp91phox indicates elevated expression of this subunit, while increased abundance of sarcolemma‐localized cytoplasmic subunits (i.e., p67phox, p47phox, and rac‐1) characterized assembly of the Nox2 complex (Figure 3). Unloading‐induced Nox2 assembly and activity in the soleus were abrogated by losartan, suggesting that AT1R triggers activation of Nox2 during disuse.

Skeletal muscle Nox activity increases due to stretch and contractile activity in both healthy (Pearson et al., 2014) as well as dystrophic muscle (Whitehead et al., 2010). In addition, elevated expression of Nox2 subunits and Nox activity has also been observed in atrophic plantaris muscle in response to myocardial infarction (Bechara et al., 2014). Moreover, our current findings are consistent with previous observations (Lawler et al., 2014), which demonstrated that hindlimb unloading upregulates protein expression and activation of sarcolemma‐localized Nox2 subunits. In concert with those findings, Bhattacharya et al. (Bhattacharya et al., 2014) also observed increased Nox activity in gastrocnemius muscles from denervated mice. Together, these data indicate that Nox2 and Nox‐derived superoxide are responsive not only to elevated activity (i.e., skeletal muscle contraction), but also to prolonged periods of inactivity (i.e., disuse) as well, and appear to trigger the general remodeling process.

Indeed, our current observation, that administration of the AT1R blocker losartan reduced the unloading‐induced increase in superoxide and Nox2 subunits (Figure 4), agrees with findings from previous studies. While the AT1R – Nox connection has been shown previously in a variety of tissues, including skeletal muscle (Sukhanov et al., 2011), this study is the first to report this connection during disuse atrophy. For example, L6 myotubes treated with AngII displayed increased Nox activity and subsequent ROS production, effects that were mitigated by the Nox inhibitor, apocynin, and the AT1R antagonist losartan (Wei et al., 2006). Furthermore, elevated ROS levels in skeletal muscle were detected in rats treated with AngII, an effect that coincided with an upregulation of gp91phox (Zhao et al., 2006).

In relation to skeletal muscle wasting, Semprun‐Prieto et al. (Semprun‐Prieto et al., 2011) observed that AngII‐induced atrophy was linked to Nox2‐induced superoxide production, as both ROS production and atrophy were prevented in p47phox^−/−^ mice (Semprun‐Prieto et al., 2011). Additional evidence demonstrated that mitochondrial ROS (Tabony et al., 2011) and mitochondrial dysfunction (McClung et al., 2009) are increased following AngII administration. However, Tabony et al. (2014) found that inhibition of mitochondrial‐derived ROS did not alter the degree of AngII‐induced skeletal muscle wasting, suggesting that AngII atrophic signaling is dependent upon Nox2 rather than mitochondrial ROS production. However, our data suggest that AT1R‐induced activation of Nox2 during unloading in skeletal muscle may be independent of blood angiotensin II levels. AT1R may serve a mechanoresponsive role instead.

Prolonged periods of disuse have been shown to cause dislocation of nNOSµ away from the sarcolemma (Lawler et al., 2014; Llano‐Diez et al., 2012; Sandona et al., 2012). Whether or not disuse leads to a reduction in nNOSµ expression and disruption of the dystrophin‐glycoprotein complex remains controversial (Lomonosova et al., 2011; Suzuki et al., 2007). Furthermore, it is not currently known whether dislocation of nNOSµ leads to rapid degradation of cytoplasmic nNOSµ. Recently, Vitadello et al. (Vitadello et al., 2014) also observed dislocation of nNOS at the sarcolemma, an effect mitigated by curcumin and overexpression of stress response protein grp‐94. Our research group previously reported that 54 hr of hindlimb unloading leads to nNOSµ translocation, concomitant with a rise in sarcolemma‐associated oxidative modifications and abrogated by a superoxide dismutase/catalase mimetic (EUK‐134) (Lawler et al., 2014). Together these findings support that elevated ROS is a causal effector of nNOSµ translocation (Lawler et al., 2014). As postulated in this study, 7 days of unloading resulted in dislocation of nNOSµ, as determined via immunofluorescence of soleus muscle cross‐sections and western blotting following subcellular fractionation (Figure 4). As hypothesized, losartan treatment throughout the unloading period offered significant protection against the unloading‐induced nNOSµ alterations in nNOS protein abundance in the membrane fraction. Furthermore, losartan produced nearly full protection of nNOS activity, as determined using NADPH‐diaphorase staining (Figure 4).

Prolonged mechanical unloading consistently triggers a partial shift from Type I to Type II fiber type, as shown here. Intriguing findings indicate that NOS, and resultant release of nitric oxide (•NO), are involved in the regulation of type I myosin heavy chain expression ((Sellman et al., 2006; Suwa et al., 2015)). For example, a significant reduction in the percentage of type I fibers and concomitant increase in Type IIa fibers were found in the rat soleus following 8 weeks of NOS inhibition (Sellman et al., 2006). Thus, unloading‐induced nNOSµ translocation may be playing a critical role in the phenotype adaptation. In support of this notion, losartan was found to offer protection against both nNOSµ translocation and muscle fiber‐type shift.

**FIGURE 7:**
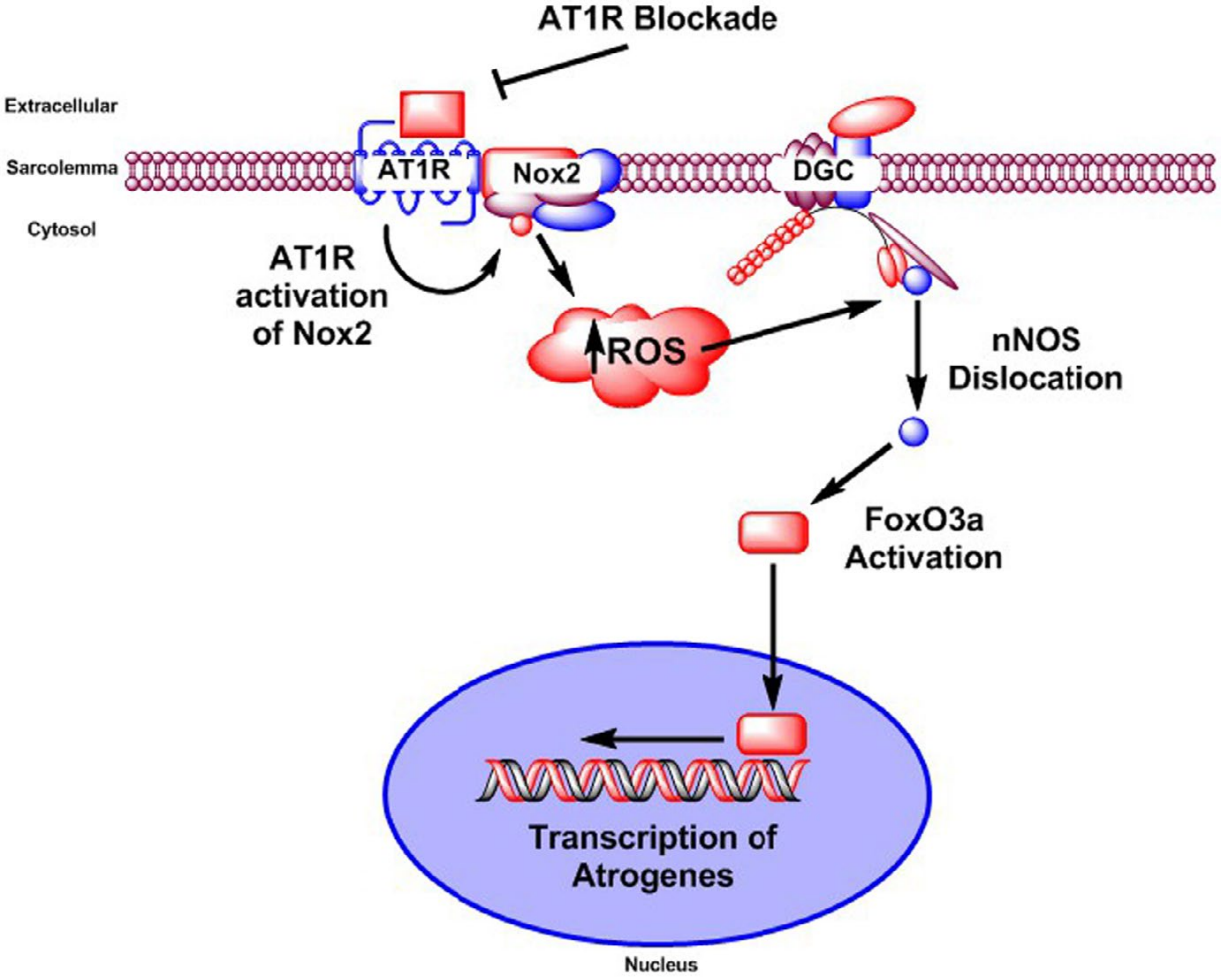
Model of the influence of AT1R activation during hindlimb unloading‐induced atrophy. Integrative model that links activation of angiotensin II type 1 receptor (AT1R) with ROS‐mediated translocation of nNOS, activation of FoxO3a and skeletal muscle atrophy

Unloading‐induced nNOSµ dislocation appears to be causal in dephosphorylation of FoxO3a (Lawler et al., 2014; Suzuki et al., 2007), thus leading to shuttling of FoxO3a to the nucleus where it acts as a transcription factor for key atrogenes: MuRF1 and MAFbx/Atrogin 1 (Suzuki et al., 2007). Indeed, expression of these two ligases is promoted by FoxO3a (Schiaffino et al., 2013). Activation of FoxO3a is sensitive to sarcoplasmic nNOS‐derived •NO (Suzuki et al., 2007), and can be inhibited by Akt (Schiaffino et al., 2013). Consistent elevation of FoxO3a was observed, detected by both DAPI‐localized immunofluorescence and nuclear fraction abundance with HU, an effect abrogated by losartan (Figure 5). This suggests that AT1R – Nox2 signaling contributes to activation of FoxO3a during mechanical unloading in the rat soleus. Dephosphorylation of FoxO1 and FoxO3a proteins, and associated transcription of the E3 ligases MuRF1 and MAFbx/Atrogin 1, have been observed in AngII treated mice (Tabony et al. 2011, 2014). Our findings suggest that AT1R is upstream of an atrophic pathway involving a pathway triggering Nox2, ROS, nNOSµ dislocation, and thus FoxO3a activation during mechanical unloading (Figure 7).

In addition to the transcription factor actions of FoxO3a during disuse, recent research has implicated the tumor suppressor p53 in the early stages of disuse atrophy. For instance, p53 protein content is elevated in the soleus during the first 6 hr of unloading (Ferreira et al., 2008), and within 3 days of disuse (Fox et al., 2014). However, it was unknown whether this occurrence persists beyond 3 days. In this study, nuclear p53 protein was significantly elevated following 7 days of unloading (Figure 6). However, unlike FoxO3a observations, losartan did not ameliorate the unloading‐induced rise in nuclear p53. Previous studies using cancer cells reported that losartan had no effect (Xiao et al., 2015), or led to an increase (Gong et al., 2010), in p53 expression. Interestingly, active p53 has been associated with promoting local renin‐angiotensin system (RAS) activity in cardiac myocytes in response to stretch (Leri et al., 1998). Local RAS signaling has also been observed in C2C12 skeletal muscle cells in response to stretch (Johnston et al., 2011), but the authors did not examine p53 involvement. Whether p53 enhances local RAS signaling in vivo or in response to disuse in skeletal muscle remains unknown. Given that p53 suppressed spermine oxidase (Bongers et al., 2015), it is possible that p53 acts upon a metabolic or nutrient sensing pathway, therefore contributing to disuse atrophy. Thus, AT1R – Nox2 signaling may be integrated into an independent mechanotransductive pathway, rather than a p53 – p21 – spermine oxidase nutrient sensing and signaling pathway previously described by Adams and colleagues (Ham et al., 2015).

### 4.1 Study limitations

While our data show that losartan administration limited soleus muscle remodeling in response to one week of hindlimb unloading, a few limitations should be considered. An increase in serum Ang II concentration was not observed in this study, which may have been due to the specific time point of serum collection at the end of the 7‐day hindlimb unloading protocol. It is possible that circulating or secreted Ang II peaks earlier during the unloading period, followed by a return to baseline levels. Future studies should include baseline and time‐course analysis of circulating Ang II concentrations from individual animals (rather than pooled samples as performed in this study) in response to rodent tail‐traction hindlimb unloading. Additional focus should be put toward evaluating the ligand‐dependent and ligand‐independent/mechano‐activation of AT1R during muscle unloading and disuse. Further examination of the AT1R subtypes and their response to unloading should also be considered. While humans have only one type of AT1R, rodents have two subtypes – AT1aR and AT1bR (Guo et al., 2001). It is still unclear which subtype plays a larger mechanosensitive role in resistance arteries (Blodow et al., 2014; Hong et al., 2016; Pires et al., 2017; Schleifenbaum et al., 2014), and it is not currently known whether the subtypes have similar functions in skeletal muscle fibers.

## 5 CONCLUSION

Our findings demonstrate that AT1R blockade during 7 days of hindlimb unloading partially prevented hindlimb unloading‐induced alterations in soleus muscle morphology (*summarized in* Figure 7). Indeed, disuse‐induced reductions in fiber CSA and shift in fiber type were limited by daily losartan administration. Additionally, disuse‐associated elevation in ROS was attenuated by losartan treatment, linked due to a reduction in assembly and activation of Nox2. Furthermore, nNOSµ dislocation from the sarcolemma due to prolonged disuse was partially prevented by losartan, as was the reduction in sarcolemmal nNOSµ activity. Antagonism of AT1R during unloading ameliorated the rise in nuclear content of FoxO3a, but not p53. However, the effects of unloading and AT1R blockade were independent of serum levels of angiotensin II.

### 5.1 Perspective and significance

To our knowledge, our study is the first to examine the effectiveness of losartan treatment on disuse‐induced skeletal muscle remodeling with administration commencing less than one week prior to the onset of disuse. Still unknown is whether losartan would be capable of offering protective effects if administration began after the onset of disuse. Protection against unloading‐induced muscle fiber atrophy and reduction in muscle quality is a critical biomedical issue with broad clinical application to spaceflight, casting, bedrest, orthopedic injuries, denervation and other effectors of skeletal muscle disuse. For example, skeletal muscle mass can decrease by 25%–30% in a bedridden ICU (intensive care unit) patient or with casting (Ham et al., 2015; Koukourikos et al., 2014). The data presented here further support the role of AT1R blockade in limiting or possibly delaying disuse atrophy, providing additional rationale for the usage of a cost‐effective and FDA‐approved pharmaceutical agent during prolonged periods of disuse (i.e., casting, bedrest, spaceflight).

## CONFLICT OF INTEREST

None declared.

## AUTHOR CONTRIBUTIONS

Author contributions: J.M.H. and J.M.L. conception and design of research; J.M.H., M.M.G., K.R.F., V.G., Y.L., and MSL developed applied technology and performed experiments; J.M.H. and J.M.L. analyzed and interpreted results of experiments; J.M.H. and J.M.L. prepared figures; J.M.H. drafted manuscript; J.M.H. and J.M.L. edited and revised manuscript; J.M.H., M.M.G., K.R.F., V.G., Y.L., and J.M.L. approved the final version of manuscript.
